# The Impact of a City-Level Minimum-Wage Policy on Supermarket Food Prices in Seattle-King County

**DOI:** 10.3390/ijerph14091039

**Published:** 2017-09-09

**Authors:** Jennifer J. Otten, James Buszkiewicz, Wesley Tang, Anju Aggarwal, Mark Long, Jacob Vigdor, Adam Drewnowski

**Affiliations:** 1 Environmental and Occupational Health Sciences, Center for Public Health Nutrition, University of Washington, Seattle 98195, WA, USA; 2 Epidemiology, Center for Public Health Nutrition, University of Washington, Seattle 98195, WA, USA; buszkiew@uw.edu (J.B.); tangw@uw.edu (W.T.); anjuagg@uw.edu (A.A.); adamdrew@uw.edu (A.D.); 3 Daniel J. Evans School of Public Policy and Governance, University of Washington, Seattle 98195, WA, USA; marklong@uw.edu (M.L.); jvigdor@uw.edu (J.V.)

**Keywords:** minimum wage, market basket, food cost, supermarkets, food price

## Abstract

*Background*: Many states and localities throughout the U.S. have adopted higher minimum wages. Higher labor costs among low-wage food system workers could result in higher food prices. *Methods*: Using a market basket of 106 foods, food prices were collected at affected chain supermarket stores in Seattle and same-chain unaffected stores in King County (n = 12 total, six per location). Prices were collected at 1 month pre- (March 2015) and 1-month post-policy enactment (May 2015), then again 1-year post-policy enactment (May 2016). Unpaired t-tests were used to detect price differences by location at fixed time while paired t-tests were used to detect price difference across time with fixed store chain. A multi-level, linear differences-in-differences model, was used to detect the changes in the average market basket item food prices over time across regions, overall and by food group. *Results*: There were no significant differences in overall market basket or item-level costs at one-month (−$0.01, SE = 0.05, *p* = 0.884) or one-year post-policy enactment (−$0.02, SE = 0.08, *p* = 0.772). No significant increases were observed by food group. *Conclusions*: There is no evidence of change in supermarket food prices by market basket or increase in prices by food group in response to the implementation of Seattle’s minimum wage ordinance.

## 1. Introduction

An increasing number of cities and municipalities across the United States have raised, or are considering raising, their minimum wage with the intent of improving the well-being of low-wage workers and their families [1,2]. Only a few studies have investigated the effects of minimum wage policies on food security, diet quality, and associated health outcomes, such as obesity and diabetes [3,4,5,6,7,8]. In addition, potential mediating factors of the relationships between minimum wage and health, such as food prices, need to be explored in greater detail. This is a particularly salient issue for lower income households, including minimum wage workers, who have lower quality diets and are at higher risk for obesity and type 2 diabetes [9,10].

The relationship between higher minimum wage and higher food prices could be significant. The U.S. food system is the largest employer of minimum wage workers, accounting for nearly one-third of the total share of low-wage workers in the nation [11]. Policies that increase minimum wage will likely increase labor costs which may result in a higher cost of business. In turn, these increased costs may be passed through the system to consumer food prices [12]. Given that lower income households spend a higher proportion of disposable income on food, they will be more vulnerable to any increase in food costs [13]. Published studies have already shown those earning minimum or low wages struggle with purchasing diets of sufficient nutritional value for health [9,14,15,16,17]. Of particular public health concern is the potential detrimental impact that added food costs could have on diet quality due to the typically higher price of healthier foods [16,17,18].

The magnitude of such effects may also vary based on the size of a minimum wage increase and whether it is experienced at a federal or local level. One study, conducted in 2000, modeled a $0.50 increase, separately for the 1992 and 1997 federal minimum wage, on food prices and concluded that small increases in the federal minimum wage would exert a less than 1% increase in food prices, even when the full labor prices were passed through to food consumers [19]. However, there was indication by study authors that larger minimum wage increases could result in greater food price increases [19]. Another study, conducted in 2012, forecasted that a 33% increase to federal minimum wage would increase retail grocery store food prices, on average, by less than 0.5% per year [11]. Both of these studies modeled a one-time increase on the federal minimum wage and used simulated rather than primary data. It is unknown whether wage increases that are larger, more localized, or phased-in incrementally over multiple consecutive years would produce different results.

In June 2014, the City of Seattle passed an ordinance mandating a $15 hourly minimum wage for businesses; it was enacted on 1 April 2015 [20,21]. The implementation of this policy is occurring over several years, with wage increases annually, until $15/hour is reached—a level that will be achieved between 2019 and 2021, depending on the size of the business and whether they offer medical benefits to their employees. This local minimum wage policy is providing an opportunity to prospectively collect and examine data on local food prices in response to real-time and incremental increases in wages over multiple years as the policy is phased in.

The primary purpose of this study is to examine immediate and short-term effects of Seattle’s minimum wage policy on local supermarket food prices after enactment of the policy. Because the policy was only targeted for businesses based in the City of Seattle, we were able to include in our sample both supermarket chains affected by the policy in Seattle and nearby, same-chain supermarkets not directly affected by the policy in King County. To assess the public health implications of potential differential price changes on specific items, such as fruits and vegetables, we also conducted a secondary analysis of the data by food group.

## 2. Methods

### 2.1. Study Design

The Seattle Minimum Wage Ordinance was adopted in June 2014 and designed such that minimum wage increases will be phased in variably between 2015 and 2019–2021 [20,22]. The policy phase-in schedule is based on both the size of the business and whether the business contributes to health insurance benefits for its workers [20,21]. As of its initial phase-in on 1 April 2015, the city’s minimum wage increased from $9.47/hour to $11/hour for most large (≥500 employees nationally) and some small businesses (<500 employees) and $10/hour for other small businesses. On 1 January 2016, the city’s minimum wage increased from $11/hour to $13/hour for most large (≥500 employees nationally) and some small businesses (<500 employees). Our study used a pre-post research design to evaluate these first two phase-ins. Supermarket price data were collected from affected and unaffected supermarkets in Seattle and King County at three time points: baseline, which was 1-month before the enactment of the ordinance (March 2015); follow-up, which was 1-month after enactment of the ordinance (May 2015) and the initial phase-in; and follow-up 2, which was 1-year after enactment of the ordinance (May 2016), a seasonal match to follow-up 1, and after the second phase-in.

### 2.2. Store Selection

The ordinance applied to 19 supermarket chains in Seattle (78 individual stores) as identified through food establishment permits provided by Public Health-Seattle and King County [9,17,23]. We narrowed the sample to six large supermarket chains inside Seattle (the “intervention” group) and six same-chain supermarkets outside Seattle but in King County (the comparison group) that were in operation during the study period. This decision was based on budget constraints and three criteria: (1) the chain had locations both inside Seattle and in surrounding King County (n = 13 of 19 chains), (2) prior research showing that, in a representative sample of Seattle and King County residents, 65% named these chains as their primary food source (n = seven of 19 chains) and, (3) to incorporate variability in market basket cost, from low price to high price [23]. The latter was determined using data from a 2009 study on market basket prices of supermarkets in King County, which illustrated that prices for the same market basket could range by store chain from $218 to $406 [23]. In addition, the six chains included in the sample represented 50 out of the 78 individual Seattle stores impacted by the ordinance. The locations of individual stores within each chain were then selected based on their proximity to lower income neighborhoods, based on methodology from previous studies [9,17,23]. Four of the six supermarket chains had employee union representation.

### 2.3. CPHN Market Basket

The use of a market basket to assess food price is common [9,16,17,23,24]. The federal government uses the USDA Thrifty Food Plan (TFP) market basket of foods to calculate the minimal cost of a nutritionally balanced diet to inform food assistance programs and the U.S. Department of Labor uses a market basket of foods, which includes the majority of foods in the TFP, to calculate a monthly indicator of national food prices and to track inflation as a component of the Consumer Price Index (CPI) [24,25]. For the present study, prices were assessed using a custom-designed market basket of 106 foods developed by the University of Washington Center for Public Health Nutrition (CPHN) in 2009 and implemented in prior studies [9,16,17,23,26]. The CPHN market basket is a combined and condensed version of prior TFP and CPI market baskets, augmented to incorporate more nutritious foods; at the time the CPHN basket was created, the TFP and CPI baskets contained 54 and 140 foods, respectively [26]. The CPHN market basket overcomes some of the limitations of the prior TFP and CPI market baskets [24,27,28,29,30]. Specifically, the TFP may not reflect the typical U.S. food preferences or eating habits and the CPI is thought to over-represent certain foods (e.g., it includes 24 forms of beef, 14 forms of pork) that are frequently purchased or lack foods thought to be integral for a healthy diet. The CPHN market basket reduces multiple versions of foods (e.g., reducing milk options from five to three by including only regular fat, 2%, and skim in half-gallon container sizes) and supplements the market basket with nutrient-rich foods [31]. The latter is achieved by using the U.S. Dietary Guidelines to incorporate recommended foods for health (e.g., brown rice, whole wheat bread) and queried foods from the Behavioral Risk Factor Surveillance System (BRFSS), such as such as salads, fruits and vegetables (e.g., apples, oranges, broccoli, carrots) [32]. Each food item was also assigned to a food group based on USDA dietary guidelines categories: “meats, beans, and proteins”, “cereals and grains”, “fruits”, “vegetables”, “dairy”, “sugar and sweets”, “fats and oils”, and “other beverages” [33]. Thus, the modified market basket includes an assortment of commonly eaten foods, as well as nutrient-rich foods recommended for a healthy diet.

Trained staff followed standard protocols to collect data on availability and prices of market basket foods at each supermarket location [16,17,23]. Every attempt was made to locate items in the market basket. Whenever items were unable to be located, a store employee was called upon for assistance in locating the item. If the item still could not be found, the closest approximation for the specified item was chosen. For example, if fresh cherries were not available, frozen cherries were substituted. Data collection required an average of 2 hours per store in order to provide adequate time to record purchasable sizes and prices, along with time spent speaking with employees when assistance was needed.

Collected prices were based on medium sizes at the time of data collection. For every store visited, the lowest priced item available was recorded; this was often the store-branded item. Sale prices, specials, coupons, and/or membership discounts were not included in data collection. Package sizes were standardized to a common unit when the purchasable form varied. For example, in some stores produce was sold by the item while in other stores by weight. The total price of the CPHN market basket, after the standardization of units, was the sum of the prices of all 106 component foods and beverages.

### 2.4. Statistical Analysis

Missing data were identified and examined. In some cases, an individual item was missing between time points, within a time point, or an item might be available at one store chain location but not the other store location. There were a total of 12 (0.9%) market basket items out of 1272 items missing at the baseline and follow-up 1 data collections. These were the same set of 12 missing items at both time points. At follow-up 2, there were 21 missing (2%) market basket items. Missing data were assumed to be missing at random. Missing items between baseline and follow-up 2 (n = 12) and follow-up 1 and follow-up 2 (n = 10) were dropped from the analyses. Descriptive statistics were used to average market basket costs by store chain and location (i.e., Seattle vs. King County) and average food group item-level prices by store chain and location. Unpaired t-tests were used to determine differences in total market basket costs and in food group item-level prices between same-chain stores by location at each time point and by store over time. Paired t-tests were used to detect differences within a store chain, averaged across Seattle and King County locations, across time. A complete-case, multi-level, linear difference-in-differences model was used to detect the changes in the average market basket item food prices that could be attributable to the minimum wage ordinance:

*Price_ijkt_* = *α_j_* + *β_k_Seattle_k_* + *γ_1_Post1_t_* + *γ_2_Post2_t_* + *δ_1_Post1_t_ × Seattle_k_* + *δ_2_Post2_t_ × Seattle_k_* + *𝜀_ijkt_* (1)

where *Price_ijkt_* is the estimated mean price for item *i* at store *j* in region *k* (i.e., affected (Seattle) or unaffected (King County) stores), at time *t*. *α_j_* is a store-level random effect. *Seattle_k_* is an indicator that equals one for Seattle stores and zero for King County stores, and *β_k_* captures differences in mean item-level prices across regions. *Post1_t_* are *Post2_t_* are indicator variables that equals one for prices measured in the first and second follow-up periods, respectively, and *γ_1_* and captures *γ_2_* differences in mean item-level prices across time relative to the baseline period. *Post1_t_ × Seattle_k_* and *Post2_t_ × Seattle_k_* equal one only for Seattle stores in the follow-up periods 1 and 2, and *δ*_1_ and *δ_2_* capture the treatment effects (i.e., the difference in prices in Seattle in the post-policy periods that cannot be explained by region and time effects, and thus appears to be attributable to the minimum wage ordinance). *𝜀_ijkt_* is the idiosyncratic error. This model was estimated with robust standard errors clustered at the store level.

The key identifying assumption in all difference-in-differences models is that time-trends in the outcome variable in the treated and control regions would be parallel in the absence of a policy change in the treatment region. In our case, the parallel trends assumption holds that price changes in King County stores, which are estimated with the coefficients on *Post1_t_* and *Post2_t_*, are good counterfactuals for price changes that would have occurred in Seattle stores in the absence of the minimum wage ordinance. Thus, *δ_1_* and *δ_2_* reflect the treatment effect as they capture deviations from parallel trends (i.e., the change in prices in King County stores as given by *γ_1_* and *γ_2_*).

To determine whether there were item-level price changes occurring within food groups, separate linear difference-in-differences models (Equation (1), above) were analyzed stratified by the eight food group categories. In this case, each estimate provides the mean difference in item-level price, as previously mentioned, but within each specified food group All analyses were conducted using Stata 14 (Stata Corporation, College Station, TX, USA) [34]. An *α* level of 0.05 was used alongside clustered robust standard errors (SE) to determine statistical significance.

## 3. Results

### 3.1. Total Market Basket Results

As shown in Table 1, differences across locations in total market basket cost ranged from –$4.24 to $8.43 across the six chains at baseline; from –$2.51 to $6.82 at follow-up 1; and, from –$13.92 to $8.55 at follow-up 2. There were no significant differences at any time point in the cost of the market basket between stores by location.

Table 2 displays the differences in market basket costs within a store chain, averaged across Seattle and King County store locations, across time.

Differences in averaged market basket price ranged from −$14.70 to $6.67 from baseline to follow-up 1, −$28.38 to $14.66 from follow-up 1 to follow-up 2, and −$23.55 to $16.94 from baseline to follow-up 2. There was a significant change in average market basket price from follow-up 1 to follow-up 2 for store chain 1 (*p* = 0.009). No other significant changes were observed across time for any other store chain.

**Figure 1:**
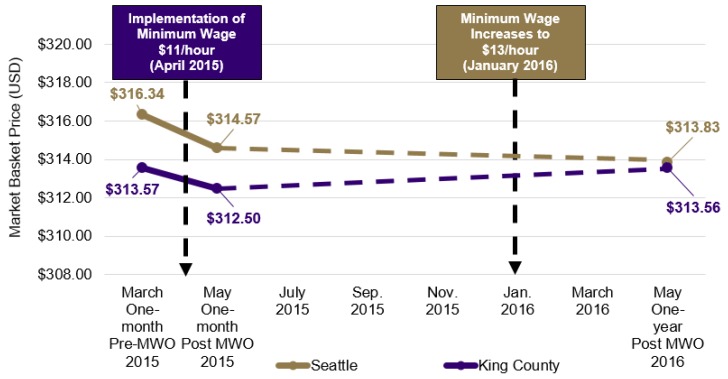
Average market basket cost before and after two phases of minimum wage law implementation in affected Seattle as compared to unaffected King County. MWO = minimum wage ordinance. Dashed lines indicated periods where data were not collected.

As shown in Figure 1, the CPHN market basket had an average cost of $316.34 at baseline, $314.57 at follow-up 1, and $313.83 at follow-up 2 in Seattle as compared to $313.57, $312.50, and $313.56, respectively, in King County. The average market basket cost declined very slightly in Seattle over time but remained relatively constant in King County. There were no significant differences in overall market basket or item-level costs at one-month (−$0.01, SE = 0.05, *p* = 0.884) or one-year post-policy enactment (−$0.02, SE = 0.08, *p* = 0.772).

### 3.2. Food Group Results 

**Figure 2:**
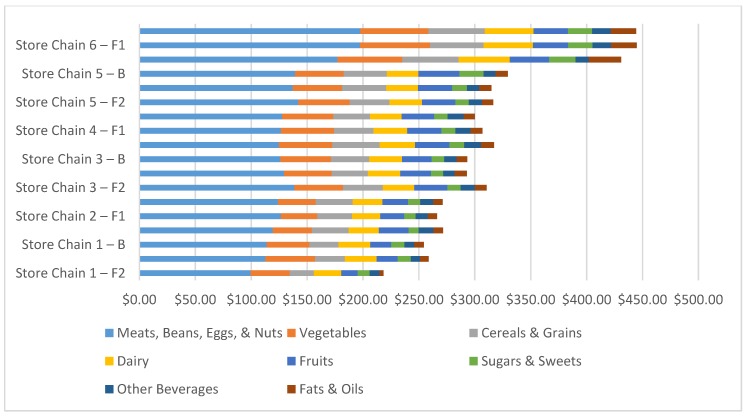
Total market basket prices broken down by food group, averaged by store chain, at baseline, follow-up 1, and follow-up 2. B = Averaged price across King County and Seattle stores, within store chain, at March 2015 Baseline. F1 = Averaged Price across King County and Seattle stores, within store chain, at May 2015 Follow-up. F2 = Averaged Price across King County and Seattle stores, within store chain, at May 2016 Follow-up.

As with the total market basket costs, there were no significant differences at any time point in food group costs in same-chain stores by location. Thus, food group cost comparisons by store chain are shown at baseline, follow-up 1, and follow-up 2 in Figure 2. Consistently, the largest share of market basket cost was meats, followed by vegetables, and then cereal and grains or dairy, across all locations at baseline and follow-up.

Overall and food group stratified, multi-level, linear difference-in-differences model results for the mean change in item-level price across Seattle and King County stores and time are shown in Table 3. Overall, there was a non-significant, item-level mean price change of −$0.01 (SE = 0.05) from baseline to follow-up 1 and −$0.02 (SE = 0.08) from baseline to follow-up 2 in Seattle supermarkets compared to King County supermarkets.

The largest observed price changes by food group between any two time points in Seattle chain supermarkets, compared to King County super markets, was in “other beverages” with a decrease of $0.55 (SE = 0.51) from baseline to follow-up 2 and in “fats and oils” with an increase of $0.28 (SE = 0.17) from baseline to follow-up 1. There were no significant differences between Seattle and King County stores over time overall or within any food group strata with the exception of a decrease of $0.22 (SE = 0.09, *p* = 0.017) in “other beverages” from baseline to follow-up 1.

## 4. Discussion

This study examined the effect of Seattle’s minimum wage policy on supermarket food prices after initial phase-in of the policy to $11/hour and, one-year later, when the minimum wage was increased to $13/hour. Total cost of the market basket at key supermarket chains was examined, both overall and by food groups. To date, we have found no evidence of changes in chain supermarket food prices related to the implementation of Seattle’s ordinance. There was also no evidence of increases by food group category.

Our overall lack of observed food price changes may be due to a number of reasons. First, baseline data collection may be proximal enough in time to the 1 April 2015 ordinance enactment that supermarkets had already incorporated price changes in anticipation of wage increases. Second, it is possible that the initial increases to $11/hour and $13/hour for most large (≥500 employees nationally) and small businesses (<500 employees) may not yet be a large enough increase in payroll for these large chain national supermarkets to warrant a change in consumer prices. Alternately, the required increases might not have been as consequential in many grocery stores because wages were already at or above that level, whether because of union contracts or because the employer already had a corporate policy of having no wage below $11 as of 2014 [35]. Using administrative earnings and hours data from over 500 grocery establishments provided by the Washington Employment Security Department, we find that 14.3% of jobs in grocery stores (NAICS Code 445110) in Seattle and 29.3% in the rest of King County earned less than $11 per hour in the year preceding the passage of the Minimum Wage Ordinance. Yet, these proportions are higher when compared to all low-wage wage jobs in all industry sectors, 7.2% and 9.9% respectively, and thus the effect of the Ordinance on prices should be greater in grocery stores than in other industries. Third, food prices may be more sensitive to national and global rather than local trends. A longitudinal time analysis of food prices between 2002 and 2014 at Seattle metropolitan grocery store chains has shown that changes in retail food prices tend to track closely with changes in the Consumer Price Index [36]. In addition, localized wage increases might have more modest effects than broader wage increases because the pass-through effects may occur only at the end of the food supply chain versus more broadly throughout the supply chain. Fourth, it is possible that higher minimum wages reduced employee turnover, which, in turn, improved worker productivity, thereby negating any detrimental effects of minimum wage [37,38].

As states and large cities consider policies and initiatives focused on wage and income supplementation in an attempt to improve the well-being of low income individuals and families, it is important to consider the effects of such efforts beyond immediate wage and income impacts, such as in public health outcomes. Currently, the evidence base regarding the effects of minimum wage on public health outcomes is limited and with little to no primary data collected on potential mediators, such as supermarket food price. Researchers have examined some diet-related outcomes, such as obesity and food security, in relation to minimum wage—hypothesizing that increases in the minimum wage could affect one’s ability to purchase additional goods or a higher quality of food, thus benefiting their health. This evidence is mixed. Two studies examining the effects of the declining value of the minimum wage on obesity rates at a population level found that a decrease in real minimum wage was associated with increased BMI (Body Mass Index ) and obesity rates [7]. One study evaluating an increase in minimum wage found that an increased minimum wage was associated with a reduction in BMI and obesity rates; however, this trend was reversed when it accounted for state-level time trends in BMI and obesity [3]. A third study found no association between minimum wage and BMI in low-income households [5]. A recent USDA study, which examined increased SNAP benefits (by an average of 17%) on food spending and insecurity, found that after households received additional SNAP benefits their inflation-adjusted food spending increased and their food insecurity declined by 2.2% [8,39].

The relationship between food prices and minimum wage is apparent. First, increased labor prices to businesses might be passed through to food prices [12]. Second, increases in family and individual incomes might alter one’s ability to afford food, including higher quality food; and, the increased demand for food might drive up prices. In the related area of fast food prices, evidence is mixed. A 1992 study found no impact of federal minimum wage increases in 1990 (from $3.35 to $3.80) and 1991 (from $3.80 to $4.25) on the cost of a full meal (fries, soda, and main course) at 3 fast food chains in Texas [40]. A more recent study looking at a federal minimum wage increase to $15/hour for food service workers in fast food restaurants found an estimated 4.3% increase in prices at those restaurants [41]. Another study, which assessed an increase in federal minimum wages on three fast food products (i.e., burgers, fried chicken, and pizza), found that a 33% increase in the federal minimum wage (from $7.25 to $10.10) would be passed through to consumers via a small 3% increase in the price of these products (e.g., a $3.77 burger would increase in price by 10 cents) [42]. Finally, a study examining San Jose, California’s 2013 wage increase from $8 to $10/hour on fast food prices found that the cost of the minimum wage increase was absorbed by price increases [43]. Conversely, a 1994 Card and Krueger study found that increases in fast food meal prices were similar regardless of differing initial wage rates, concluding that price changes were not simply related to wage pass-through effects [44]. Little is known about how an increase in minimum wage and thus labor wages will affect primary food shopping sources such as supermarkets, especially salient for low-income shoppers who spend a greater proportion of their income on food [16,17,23]. Our study attempts to provide information on supermarket food prices and has relevance to researchers looking at the relationships between wage and health outcomes via food prices and decision makers considering the bigger picture of public health impacts of policies focused on wage and income supplementation.

This study contributes to the limited research on the effects of local policies on food prices. Strengths of the study include the use of primary and prospective data collection at a local level before and after enactment of the ordinance at major supermarket chains and the use of an established method. This study also has several limitations. First, food quality was not captured in this study because it is difficult to measure objectively. One person’s subjective evaluation of “fresh” fruit may be another’s assessment of “overly-ripe”. This may explain some of the differences in total market basket price across store brands. For example, supermarket chain 6 was observed to carry more items that are often considered higher quality, such as those labelled as organic, grass-fed, farmer friendly, hormone-free, and antibiotic-free [17]. In addition, some stores may carry more locally sourced produce than others, which is shown to be less expensive and may be disproportionately impacted by changes in minimum wage due to upstream increases in labor prices [45,46]. However, we hoped to minimize these differences at the store level by selecting same-chain supermarkets that may source and provide similar selections. Second, data on the variability in price due to sales, coupons, store specials, and/or membership discounts was not collected. In addition, data were collected on the lowest priced item available rather than tracking the same brand over time. It is possible that supermarkets could absorb some of the price associated with increases in payroll by reducing the frequency of sales or the size of discount or by increasing the price of their store brands, which would not have been captured by this study. Data collected on the lowest price item available rather than the same brand over time may have biased our findings towards the null. Third, this study’s primary focus was about economic access, as opposed to physical access, toward attaining food, particularly foods that comprise a healthy diet. While the supermarkets selected were near major highways and arterials, no assessment was made on the accessibility by public transportation or other physical barriers that pertain to food acquisition. In addition, the selection of supermarkets based on their proximity to low income neighborhoods is a highly crude measure of the socioeconomic status of the patrons who shop there as it has been shown through previous research that price, rather than proximity, is the major driver for where individuals purchase their groceries [23]. To offset this, we oversampled supermarkets in the lower-price ranges. Finally, the study sample did not include bulk retailers, specialty stores, ethnic stores, convenience stores, farmers’ markets, online deliveries, and other food retailers. This was purposefully done in order to apply the market basket tool, with standardized sizes, for comparison across all stores.

## 5. Conclusions

This study serves to provide both data for comparison and as a methodology that other municipalities might use to examine the impact of such a policy on their local supermarket food prices. This study found no evidence of increases in supermarket food prices by market basket or food group in response to the implementation of Seattle’s minimum wage ordinance. The lack of minimum wage ordinance pass through effect to consumer food prices may be encouraging as the ordinance is designed to improve the lives of low income households who often struggle to afford high quality diets and have a higher prevalence of chronic disease, such as obesity and type 2 diabetes. Future supermarket price data collection will serve to evaluate longer-term exposure to Seattle’s minimum wage and an increase to $15/hour. Additional research is warranted in other economic contexts where municipalities are also increasing their minimum wage.

## Figures and Tables

**Table 1:** Comparison of total market basket price, by supermarket chain and location, at baseline, follow-up 1, and follow-up 2.

Store Chain	Seattle Store Market Basket Price	King County Store Market Basket Price	Difference in Price between Locations	*p*-value ^a^
Baseline				
Store Chain 1	257.34	251.73	5.61	0.841
Store Chain 2	272.04	270.43	1.61	0.957
Store Chain 3	297.43	289.00	8.43	0.780
Store Chain 4	297.99	302.23	−4.24	0.891
Store Chain 5	333.37	325.51	7.86	0.827
Store Chain 6	442.88	445.62	−2.75	0.949
**Follow-up 1**				
Store Chain 1	262.18	255.36	6.82	0.797
Store Chain 2	265.04	267.55	−2.51	0.935
Store Chain 3	292.14	293.70	−1.56	0.960
Store Chain 4	308.90	304.68	4.22	0.888
Store Chain 5	314.20	315.29	−1.09	0.973
Store Chain 6	447.98	441.51	6.47	0.883
**Follow-up 2**				
Store Chain 1	227.47	218.92	8.55	0.679
Store Chain 2	270.68	263.12	7.56	0.750
Store Chain 3	307.57	308.50	−0.93	0.977
Store Chain 4	310.64	324.56	−13.92	0.622
Store Chain 5	315.22	311.95	3.27	0.918
Store Chain 6	442.20	445.39	−3.19	0.777
**All stores averaged**				
Baseline	316.84	314.09	2.76	0.844
Follow-up 1	315.07	313.02	2.06	0.882
Follow-up 2	310.83	307.65	3.18	0.808

^a^: Unpaired t-tests used to detect differences between locations.

**Table 2:** Comparison of total market basket cost, by time store chain, averaged across locations, at baseline, follow-up 1, and follow-up 2.

Store Chain	Average Market Basket Price Baseline	Average Market Basket Price Follow-up 1	Average Market Basket Price Follow-up 2	Change B to F1	Change F1 to F2 ^a^	Change B to F2 ^a^
Store Chain 1	$254.53	$258.77	$218.28	$4.24	−$28.38^†^	−$23.55
Store Chain 2	$271.24	$266.30	$271.54	−$4.94	$2.35	−$2.59
Store Chain 3	$293.22	$292.92	$310.60	−$0.30	$14.66	$14.47
Store Chain 4	$300.12	$306.79	$317.05	$6.67	$10.26	$16.94
Store Chain 5	$329.45	$314.75	$316.44	−$14.70	$2.63	−$12.42
Store Chain 6	$444.24	$444.75	$430.88	$0.50	$3.63	$4.13
Overall	$315.47	$314.04	$310.80	−$1.42	$0.86	−$0.50

**^†^** Indicates *p*-value < 0.05. *p*-values calculated using paired t-tests to detect differences in store chains, averaged across locations, between time points. ^a^: Change calculations exclude inconsistent items between time points therefore change calculations may not equal the difference in columns. B = Averaged price across King County and Seattle stores, within store chain, at March 2015 Baseline. F1 = Averaged Price across King County and Seattle stores, within store chain, at May 2015 Follow-up. F2 = Averaged Price across King County and Seattle stores, within store chain, at May 2016 Follow-up.

**Table 3:** Overall and food group stratified linear difference-in-differences model results for the mean change in item-level price across Seattle and King County stores and time, from March 2015 to May 2016.

Mean Difference in Prices Estimate	Overall	Food Group							
		(1) Other Beverages	(2) Cereals and Grains	(3) Dairy	(4) Fats and Oils	(5) Fruits	(6) Meats, Beans, Eggs, and Nuts	(7) Sugar and Sweets	(8) Vegetables
Seattle [relative to King County] (SE)	$0.03	$0.18	−$0.01	$0.04	−$0.08	$0.08	−$0.01	$0.02	$0.06
	(0.35)	(0.55)	(0.31)	(0.29)	(0.77)	(0.26)	(0.55)	(0.49)	(0.20)
Follow-up 1 [relative to baseline period] (SE)	−$0.01	$0.01	−$0.06	−$0.04	−$0.12	−$0.05	$0.04	−$0.27	$0.07
	(0.02)	(0.07)	(0.06)	(0.07)	(0.10)	(0.11)	(0.06)	(0.17)	(0.06)
Follow-up 2 [relative to baseline period] (SE)	$0.00	$0.32	$0.05	$0.05	$0.26	$0.06	−$0.13	−$0.24	$0.04
	(0.06)	(0.41)	(0.16)	(0.08)	(0.27)	(0.13)	(0.11)	(0.24)	(0.03)
Seattle x Follow-up 1 (SE)	−$0.01	−$0.22 *	$0.07	$0.10	$0.28	−$0.01	−$0.07	$0.02	−$0.06
	(0.05)	(0.09)	(0.09)	(0.08)	(0.17)	(0.13)	(0.08)	(0.33)	(0.09)
Seattle x Follow-up 2 (SE)	−$0.02	−$0.55	−$0.01	−$0.01	$0.00	−$0.06	$0.05	-$0.02	−$0.02
	(0.08)	(0.51)	(0.20)	(0.10)	(0.41)	(0.18)	(0.15)	(0.41)	(0.04)
Observations	3776	108	498	431	143	456	1075	216	849
Number of stores	12	12	12	12	12	12	12	12	12
R² within	0.00001	0.01020	0.00087	0.00076	0.00342	0.00057	0.00036	0.00701	0.00044
R² between	0.00019	0.00299	0.00029	0.00496	0.01330	0.00988	0.00046	0.00030	0.00283
R² overall	0.00002	0.00845	0.00077	0.00138	0.00269	0.00103	0.00037	0.00576	0.00068

(SE) = Robust standard errors, clustered by store, is shown in parentheses. *p*-values come from Wald tests. * *p* < 0.05.

## Supplementary Materials

Supplementary File 1
